# Dramatically Amplified Thoracic Sympathetic Postganglionic Excitability and Integrative Capacity Revealed with Whole-Cell Patch-Clamp Recordings

**DOI:** 10.1523/ENEURO.0433-18.2019

**Published:** 2019-05-10

**Authors:** Michael Lee McKinnon, Kun Tian, Yaqing Li, Alan Joel Sokoloff, Meredith Lucy Galvin, Mi Hyun Choi, Astrid Prinz, Shawn Hochman

**Affiliations:** 1 Department of Physiology, Emory University, Atlanta, GA 30322; 2 Department of Biology, Emory University, Atlanta, GA 30322

**Keywords:** computational model, firing properties, membrane properties, mouse, paravertebral ganglia

## Abstract

Thoracic paravertebral sympathetic postganglionic neurons (tSPNs) comprise the final integrative output of the distributed sympathetic nervous system controlling vascular and thermoregulatory systems. Considered a non-integrating relay, what little is known of tSPN intrinsic excitability has been determined by sharp microelectrodes with presumed impalement injury. We thus undertook the first electrophysiological characterization of tSPN cellular properties using whole-cell recordings and coupled results with a conductance-based model to explore the principles governing their excitability in adult mice of both sexes. Recorded membrane resistance and time constant values were an order of magnitude greater than values previously obtained, leading to a demonstrable capacity for synaptic integration in driving recruitment. Variation in membrane resistivity was the primary determinant controlling cell excitability with vastly lower currents required for tSPN recruitment. Unlike previous microelectrode recordings in mouse which observed inability to sustain firing, all tSPNs were capable of repetitive firing. Computational modeling demonstrated that observed differences are explained by introduction of a microelectrode impalement injury conductance. Overall, tSPNs largely linearly encoded injected current magnitudes over a broad frequency range with distinct subpopulations differentiable based on repetitive firing signatures. Thus, whole-cell recordings reveal tSPNs have more dramatically amplified excitability than previously thought, with greater intrinsic capacity for synaptic integration and with the ability for maintained firing to support sustained actions on vasomotor tone and thermoregulatory function. Rather than acting as a relay, these studies support a more responsive role and possible intrinsic capacity for tSPNs to drive sympathetic autonomic function.

## Significance Statement

Thoracic sympathetic postganglionic neurons (tSPNs) represent the final neural output for control of vasomotor and thermoregulatory function. We used whole-cell recordings and computational modeling to provide broad insight on intrinsic cellular mechanisms controlling excitability and capacity for synaptic integration. Compared to past intracellular recordings using microelectrode impalement, we observed dramatically higher membrane resistivity with primacy in controlling enhanced tSPN excitability and recruitment via synaptic integration. Compared to reported phasic firing, all tSPNs fire repetitively and linearly encode injected current magnitude to firing frequency over a broad range. Modeling studies suggest microelectrode impalement injury accounts for differences in tSPN properties previously observed. Overall, intrinsic tSPN excitability plays a much greater role in the integration and maintenance of sympathetic output than previously thought.

## Introduction

Sympathetic postganglionic neurons (SPNs) within paravertebral chain ganglia represent a large fraction of the final output of the sympathetic nervous system. Whereas prevertebral sympathetic ganglia are typically associated with one or more visceral organs in a discrete location (celiac ganglion, superior/inferior mesenteric ganglion), thoracic paravertebral chain ganglia are associated with control of dispersed tissue systems such as vasculature, brown adipose tissue, sweat glands, and piloerector muscles (Jänig, 2006; Bartness et al., 2010). As such, the sympathetic chain can be thought of as a distribution system for sympathetic activity that spans the body. The vast majority of paravertebral postganglionic neurons in mice are adrenergic (Gibbins, 1991; Jobling and Gibbins, 1999) since sweat glands, innervated by cholinergic postganglionic neurons, are largely absent in the mouse (Lu and Fuchs, 2014).

Traditionally, thoracic SPNs (tSPNs) have been envisioned as passive followers of intraspinal preganglionic neuronal activity. By this viewpoint, postganglionic neurons fire if and only if preganglionics fire and serve as 1:1 relays that pass central commands to the periphery (Jänig, 2006). This relationship is explained by the “*n* + 1” rule, wherein postganglionic neurons receive n small synaptic inputs, and one major, always suprathreshold input which leads to firing with a high safety factor. The n smaller synaptic inputs are typically sub-threshold and infrequent, and are not thought to contribute appreciably to the firing rate (McLachlan et al., 1998; Karila and Horn, 2000; McLachlan, 2003; Wheeler et al., 2004; Rimmer and Horn, 2010). However, recent evidence from rodent sympathetic ganglia has shown that postganglionic neurons play a more active role in shaping sympathetic output (Bratton et al., 2010; Springer et al., 2015). In light of these findings, we must reconsider the role that SPNs play in synaptic integration and signal transmission.

Despite their critical importance as the final output controlling sympathetic neural commands, surprisingly little is known about the SPNs in thoracic segments (tSPNs) of the sympathetic chain. The most likely reason for this is their near inaccessibility to *in vivo* study, and the relative difficulty for *in vitro* cellular characterization. Because of this difficulty, electrophysiological properties of sympathetic neurons have been largely inferred from recordings in other mammalian paravertebral sympathetic ganglia, namely the superior cervical ganglion (SCG; Eccles, 1935; Erulkar and Woodward, 1968; Purves and Wigston, 1983; Li and Horn, 2006) and to a lesser extent the stellate and lumbar ganglia (Jänig, 1985; Cassell et al., 1986; Valli et al., 1989; Bratton et al., 2010). Compared to SCG, mouse tSPNs are smaller, have less elaborate dendritic arbors, are likely more excitable, and differ in measures of action potential (AP) shape (Jobling and Gibbins, 1999). Thoracic ganglia also contain a different subset of molecularly distinct SPN subpopulations and project to different end-organs (Jänig, 2006; Furlan et al., 2016). Unfortunately, few studies have directly characterized electrophysiological properties of thoracic ganglia (Blackman and Purves, 1969; Lichtman et al., 1980; Jobling and Gibbins, 1999). These studies used sharp microelectrodes for recordings, which likely introduce a considerable impalement injury conductance compared to whole-cell patch-clamp recordings (Staley et al., 1992; Springer et al., 2015). This injury-induced conductance alters basic membrane properties, such as input resistance and membrane time constant, which reduce recruitment and synaptic integrative actions according to classical cable theory (Rall, 2011; Springer et al., 2015). The impalement conductance introduced by microelectrode recordings can also prevent expression of repetitive firing properties (Cymbalyuk et al., 2002; Springer et al., 2015). Indeed, while it is generally thought that most paravertebral SPNs fire phasically (Cassell et al., 1986; Jobling and Gibbins, 1999; Li and Horn, 2006), whole-cell recordings in SCG support repetitive rather than phasic firing (Springer et al., 2015). Whether repetitive firing properties are predominant in tSPNs remains unknown.

The aim of the present study is to investigate the electrophysiological properties of tSPNs using whole cell recordings to more accurately characterize the cellular mechanisms that drive and modulate excitability of tSPNs. We furthermore matched recordings to a computational model to better understand how synaptic inputs and passive and active membrane properties interact to recruit neurons and generate the firing properties observed.

## Materials and Methods

### Animals

All animal procedures were performed in accordance with the Emory University Institutional Animal Care and Use Committee’s regulations and conformed to the Guide for the Care and Use of Laboratory Animals. Experiments were performed on adult (P37–P379) C57BL/6 mice (RRID:IMSR_JAX:000664). Mice were anesthetized with inhaled isoflurane and maintained or killed with urethane (intraperitoneal injection, 40 mg/kg for transcardial perfusions, ∼500 mg/kg for *in vitro* electrophysiology). Complete sedation or death was confirmed by lack of foot pinch and eye blink reflex.

### Immunohistochemistry

#### Neurotransmitter identity

Two ChAT-eGFP mice (RRID:IMSR_JAX:007902), a male and a female (P91 and P101, respectively) were anesthetized and transcardially perfused with heparinized saline (0.9% NaCl, 0.1% NaNO_2_, 10-units/ml heparin), followed by 4% paraformaldehyde (0.5 M phosphate, 4% paraformaldehyde, NaOH). Tissue was post-fixed overnight, then transferred to a 15% sucrose solution and stored at 4°C. Sympathetic chains were isolated from stellate (T1 and T2) to T12/13. Tissue was embedded (TissueTek optimal cutting temperature compound), sectioned on a cryostat (−21°C, 8-μm slice thickness), and mounted on glass slides. Tissue was washed in 0.1 M PBS for 1 h and permeabilized with PBS containing 0.3% Triton X-100 (PBS-T) overnight. Sections were subsequently incubated for 2–3 d with primary antibodies: sheep anti-tyrosine hydroxylase (TH; Millipore, 1:100, RRID:AB_90755) and chicken anti-green fluorescent protein (Jackson, 1:100). Preparations were then washed in PBS-T (3 × 30 min) and incubated for 1.5 h with secondary antibodies: Cy3 donkey anti-sheep (Abcam, 1:250) and Alexa Fluor 488 donkey anti-chicken (Abcam, 1:250). Slides were washed a final time in PBS-T (20 min), then 50 mM Tris-HCl (2 × 20 min) and allowed to dry before being coverslipped [SlowFade Gold antifade reagent with 4',6-diamidino-2-phenylindole (DAPI)]. Sections were visualized under a fluorescent microscope (Olympus BX51). Cells with visible nuclei were counted and assessed for neurotransmitter identity. Interanimal cell count variability was substantial (6494 vs 19,721 cells).

#### Cell diameter

Six C57Bl/6J mice (RRID:IMSR_JAX:000664), 5 males and one female (all ∼P60) were transcardially perfused, as above. T5 Sympathetic ganglia were isolated. Unmounted tissue was washed in PBS-T overnight. Slides were subsequently incubated for 5 d with sheep anti-TH (Millipore, 1:100, RRID:AB_90755). Preparations were then washed in PBS-T (3 × 2 h) and incubated for 3 d with Alexa Fluor 488 donkey anti-sheep (Jackson, 1:100). Slides were washed a final time in PBS-T (2 h), then 50 mM Tris-HCl (2 × 1 h). Intact ganglia were mounted on glass slides and coverslipped (SlowFade Gold antifade reagent with DAPI). TH-immunoreactive cells were visualized under a fluorescent microscope (Olympus BX51, 40× objective) using a Microfire digital camera (Optronics), and traced using Neurolucida software (MBF Bioscience, RRID:SCR_001775). Cell diameters were calculated as the arithmetic mean of minimum and maximum Feret. Diameter was only determined for cells with a discernible perimeter (176 ± 131 cells per ganglion) representing a mean 71% of the total TH^+^ cell population (range of 36–95% neurons/ganglia measured). As diameter distributions were comparable between ganglia, the possibility of sampling bias in estimated cell diameter is unlikely. Results are reported as mean ± SD.

### Electrophysiology

#### Tissue preparation

**Figure 1.**
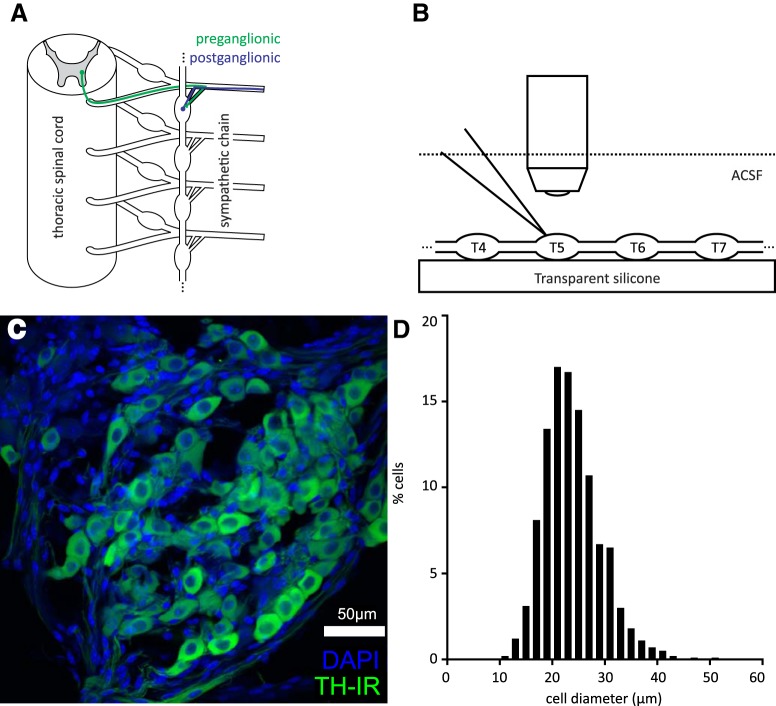
Cell size and composition. ***A***, Simplified schematic depicting the anatomic organization of preganglionic and postganglionic neurons. ***B***, Recording setup. Sympathetic chains are pinned down in a silicone chamber, superfused with oxygenated ACSF. They are then visualized under a microscope and recorded using a glass patch-clamp electrode. ***C***, Confocal slice through whole-mounted tissue showing TH immunolabeling and nuclear labeling with DAPI. Note the numerous smaller and more intensely labeled nuclei that are presumably non-neuronal cells. Scale bar represents 50 µm. ***D***, Histogram showing distribution of TH-IR cell diameters in T5 ganglia of six animals.

Mice were killed and the spinal column was quickly dissected out with sympathetic chain and spinal roots attached. Figure 1*A* provides a simplified schematic of the anatomic organization of intraspinal preganglionic and paravertebral postganglionic neurons. The remaining tissue was incubated in continually oxygenated ACSF containing collagenase (20-mg Type III per 1-ml ACSF, Worthington Biochemical Corporation) for 1.5 h. ACSF used for incubation was buffered with either bicarbonate or HEPES. No difference was observed as a result of incubation buffer. Following incubation, tissue was vortexed to remove adherent fat and washed with ACSF several times to eliminate residual collagenase. The intact sympathetic chain was removed by severing rami, and was then pinned down into a clear Sylgaard recording dish (Fig. 1*B*), through which recirculating, oxygenated ACSF was continually perfused.

##### Whole-cell recordings

Whole-cell patch recordings were obtained from postganglionic cells at room temperature. Cells were identified using an upright microscope (Olympus, BX51WI) affixed with a low-light camera (Olympus, OLY-150). Patch electrodes were pulled on a vertical puller (Narishige, PP-83) from 1.5-mm outer diameter filamented, borosilicate glass capillaries (World Precision Instruments, stock #TW150F-4) for a target resistance of 5–9 MOhm. Signals were amplified using a MultiClamp 700A and digitized at 10 kHz using a Digidata 1322A and Clampex software (Molecular Devices, RRID:SCR_011323).

We considered for analysis all cells which displayed clearly defined APs on depolarization by square current steps. Of these, cells were excluded if more than 100 pA was required to hyperpolarize a cell to −70 mV (indicative of a significant leak), if APs appeared stunted (indicative of an incomplete breakthrough), or if membrane potential was highly variable (indicative of improper seal formation). All cells which met these criteria (*n* = 35) had resting membrane potentials more negative than −50 mV and input resistances higher than 200 MΩ. All recordings were made in ACSF containing: 127.99 mM NaCl, 1.90 mM KCl, 1.30 mM MgSO_4_·7H_2_O, 2.40 mM CaCl_2_·2H_2_O, 1.20 mM KH_2_PO_4_, 9.99 mM glucose, and 26.04 mM NaHCO_3_. ACSF pH was adjusted to 7.4 after saturation with gas (95%O_2_, 5%CO_2_) at room temperature. Intracellular patch-clamp solution contained: 140.0 mM K-gluconate, 11.0 mM EGTA, 10 mM HEPES, and 1.32 mM CaCl_2_; pH was adjusted to 7.3 using KOH. Target osmolarity was <290 mOsm. In most recordings (25/39 cells), support solution was added consisting of 4.0 mM ATP and 1.0 mM GTP.

The ratio of male to female mice was ∼1:1. Recordings were taken from the right thoracic ganglia, with the majority of recordings coming from T5. The number of cells from ganglia T3 through T12 was 2, 6, 17, 3, 2, 1, 1, 0, 1, and 2, respectively. We initially assessed sex and segment related differences in cell properties. Our sample size precludes a more detailed analysis of segment-specific differences, but we were able to pool data from rostral (T3 and T4) and caudal (T5 to T12) segments (Furlan et al., 2016). Statistical tests revealed no differences with respect to sex or rostro-caudal location (*t* test, two-tailed, unequal variance, Šidák corrected α = 0.0019), so all data were pooled for additional analyses.

### Data analysis

All cellular properties were analyzed in Clampfit (Molecular Devices, RRID:SCR_011323) or MATLAB (MathWorks, RRID:SCR_001622). All parameters were estimated from a single set of current steps for each cell. This ensured that parameters for a given cell were estimated at nearly the same point in time. In current clamp mode, membrane voltage response to hyperpolarizing current steps of at least 1.5 s was fit to an exponential of the form Equation 1 using the Levenberg–Marquardt algorithm built in to Clampfit. The value of membrane time constant (τ_m_) was calculated in this manner (Golowasch et al., 2009). R_in_ was estimated by dividing maximal voltage deflection (ΔV) by the injected current (I_inj_; Eq. 2). Membrane capacitance (C_m_), a measure of total cell surface area, was estimated by dividing τ_m_ by R_in_ (Eq. 3).

(1) $$\Delta V\cdot \exp\left(-t/\tau_{m}\right)+(V_{hold}-\Delta V)$$

(2) $$R_{in}=\Delta V/I_{inj}$$

(3) $$C_{m}=\tau_{m}/R_{in}$$

Measured rheobase current (I_rheo_) was taken as the smallest long-duration (1.5 s or longer) positive current injection which elicited a single spike. In the case that an incremental increase in current elicited multiple spikes, rheobase was estimated to be the mean of the adjacent subthreshold and suprathreshold steps, e.g., if 30 pA did not elicit any spikes but 40 pA elicited several, the measured rheobase estimate would be 35 pA. to achieve a more finely-grained estimate of rheobase, we also calculated rheobase based on the equation:

$${\hat{I}}_{rheo}=\left(V_{th}-V_{hold}\right)/R_{in}$$

where V_th_ is the AP threshold, taken to be the point at which the first derivative of voltage, dV_m_/dt, begins to increase (Platkiewicz and Brette, 2010). Measured values related to AP and post-spike afterhyperpolarization (AHP) characteristics were taken from traces elicited at minimal suprathreshold current, i.e. the smallest current magnitude used which elicited at least one AP.

The parameters of the fast AHP (fAHP) varied as a function of firing rate, so analysis of fAHP properties was limited to cells which fired a single spike at minimal suprathreshold current intensity. AP amplitude was defined as the difference between the peak voltage and threshold. AP half-width is the width of the spike at half AP amplitude. fAHP amplitude was defined as the difference between peak negative voltage and steady-state voltage at rheobase current injection. fAHP half-decay is the time it takes for the fAHP to decay to half its amplitude. fAHP duration is the time between spike onset and return to baseline (Hochman and McCrea, 1994). Slow AHP (sAHP) amplitude was defined as the difference between peak negative voltage and baseline (holding voltage). sAHP half-decay is the time it takes for the sAHP to decay to half its amplitude.

Instantaneous firing rate (IFR) was taken as the inverse of the interspike interval. Maximal firing rate was the IFR for the first spike pair at the beginning of current onset. Sustained firing rate was the mean IFR for the last three interspike intervals, given they occur during the last half of the depolarizing current step. Frequency-current (ƒ-I) slope is the slope of the linear regression of the ƒ-I curve. The spike rate adaptation (SRA) ratio is defined as the ratio between the maximal and sustained firing rate at a given current injection (Venance and Glowinski, 2003; Miles et al., 2005). to directly compare firing rate across cells with variable R_in_ and ƒ-I curves, we used the sustained firing rate at twice minimal suprathreshold current injection. Current step duration was at least 1.5 s for all cells, and 3 s for the majority. Liquid junction potential was calculated to be −9.8 mV and empirically measured to be −13 mV. All values of absolute voltage (resting membrane potential, absolute threshold, peak voltage) were adjusted by −10 mV to approximately account for liquid junction potential. For example, a recorded AP peak of 30 mV would be reported as 20 mV and a recorded RMP of −60 mV would be reported as −70 mV.

### Computational modeling

#### Single neuron model

We built a conductance-based neuron model to help understand observed results in relation to their underlying biophysical mechanisms. While tSPNs do possess dendrites, their dendritic arborizations are relatively simple. We therefore assume that ganglionic cells are electrotonically compact, and that a single-compartment model can replicate all essential physiologic properties observed in experiments. All currents included in the model have been observed in rodent sympathetic ganglia (Galvan and Sedlmeir, 1984; Sacchi et al., 1995; Jobling and Gibbins, 1999; Rittenhouse and Zigmond, 1999) and transcript expression in mouse thoracic ganglia has recently been confirmed by a single-cell RNA sequencing study (Furlan et al., 2016).

The model is based on a model of bullfrog paravertebral sympathetic ganglia (Wheeler et al., 2004), which represents the most complete available computational model of a paravertebral neuron. From this model the following conductances were taken: a fast sodium current, I_Na_; a delayed-rectifier potassium current, I_Kd_; a slow and non-inactivating potassium current, I_M_; and a voltage-independent leak current, I_leak_. Additional conductances were added from models derived in other species. These include the following: a fast transient potassium current, I_A_ (Rush and Rinzel, 1995); a hyperpolarization-activated inward current, I_h_ (Kullmann et al., 2016); and a calcium-dependent potassium current, I_KCa_ (Ermentrout and Terman, 2010). I_KCa_ depends on intracellular calcium concentration, [Ca^2+^], so a model of persistent calcium current, I_CaL_ (Bhalla and Bower, 1993) and somatic calcium dynamics (Kurian et al., 2011) were added as well. Model parameters were then tuned to fit recorded data from the present study.

The membrane voltage, V, is updated according to the equation:

(4) $$C_{m}\frac{dV}{dt}=-\sum^{\text{​}}I_{i}-I_{input}$$

Membrane capacitance, C_m_, was set at 100 pF to approximate the mean in recorded neurons. Each current, I_i,_ is described by the equation:

(5) $$I_{i}=G_{i}m^{p}h^{q}\left(V-E_{i}\right)$$

where G_i_ is the maximal conductance, E_i_ is the reversal potential, and m and h are gating variables for activation and inactivation. A standard model neuron was used to replicate the majority of observed phenomena. Maximal conductances of this standard neuron are indicated in Table 1. The standard model was modified as necessary to fit individual recordings, which comprise a heterogeneous population. The reversal potentials for the various membrane currents are indicated in Table 1.

**Table 1.** Model parameters

Current	G_max_ (nS)	E_rev_ (mV)
I_Na_	300	60
I_CaL_	1.2	120
I_Kd_	2000	−90
I_M_	50	−90
I_KCa_	50	−90
I_A_	50	−90
I_H_	1	−32
I_leak_	1	−55
I_imp_*	0	−15

Maximal conductance and reversal potential for the standard model neuron used for computational analysis. *Note that I_imp_ is set to 0 nS as it is only included in simulations concerned with microelectrode impalement.

The activation and inactivation variables m and h are updated by the equation:

(6) $$\frac{dx}{dt}=\frac{x_{\infty }-x}{\tau_{x}}$$

The intracellular calcium concentration is updated by:

(7) $$\frac{d}{dt}\left[Ca^{2+}\right]=\lambda \left(-\alpha I_{CaL}-k_{CaS}\left[Ca^{2+}\right]\right)$$

where λ = 0.01 is the ratio of free to bound [Ca^2+^], α = 0.002 μM·ms^−1^·pA^−1^ is the conversion factor from current to concentration, and k_CaS_ = 0.024 ms^−1^ is the somatic [Ca^2+^] removal rate.

##### Impalement simulation

To replicate impalement injury, an additional leak conductance was added to the model to simulate microelectrode impalement. This conductance, g_imp_, was modeled as a non-selective ohmic leak channel with E_imp_ = −15 mV. The impalement reversal potential was estimated by solving the Goldman–Hodgkin–Katz equation (Eq. 8), with equal permeabilities of the three major ionic species. This estimate agrees well with estimates of impalement reversal potential in bullfrog ganglia (Brown, 1988). For analysis, model neurons were subjected to a bias current and held at −70 mV, unless otherwise stated. g_imp_ was normally set at 0 nS, and was only included where indicated for simulation of impalement.

(8) $$E_{m}=\frac{RT}{F}ln\left(\frac{P_{Na}{\left[Na^{+}\right]}_{out}+P_{K}{\left[K^{+}\right]}_{out}+P_{Cl}{\left[Cl^{-}\right]}_{in}}{P_{Na}{\left[Na^{+}\right]}_{in}+P_{K}{\left[K^{+}\right]}_{in}+P_{Cl}{\left[Cl^{-}\right]}_{out}}\right)$$

##### Synapse simulation

Synaptic input was implemented with equation:

(9) $$I_{syn}\left(t\right)=A\cdot g_{syn}\left(t\right)\cdot \left(V-E_{syn}\right)$$

where I_syn_ is synaptic current, A is conductance amplitude, and E_syn_ is the synaptic reversal potential set at 0 mV. Synaptic conductance, g_syn_, was calculated from the equation:

(10) $$g_{syn}\left(t\right)=s\cdot \left(e^{-t/\tau_{d}}-e^{-t/\tau_{r}}\right)$$

where τ_r_ and τ_d_ are the rise and decay time constants, respectively, and s is a scaling factor to normalize the amplitude to 1 nS. Equations were adapted from Springer et al. (2015). Rise and decay time constants were 1 and 15 ms, respectively, as estimated from voltage clamp recordings of spontaneous synaptic activity.

##### Code accessibility

Source code for all simulation and analysis are available online at https://github.com/pinewave/tSPN and ModelDB (Hines et al., 2004, accession #245926). Simulation and analysis scripts were written in Python 2.7.10 and executed in PyCharm (CE 2017.1.2) on macOS 10.12.3 with a 1.7-GHz processor. Scripts were also translated into MATLAB code and executed on Windows 10 with a 2.4-GHz processor. All differential equations were integrated using an Exponential Euler method with a time step of 0.1 ms (Prinz et al., 2004).

### Experimental design and statistical analysis

The present study used a descriptive design. Statistical analyses were performed in Microsoft Excel. Basic properties are presented as mean ± SD in Table 2. Correlations were determined by Pearson’s correlation coefficient, *r*. A two-tailed *t* test was used to calculate each *p* value. To control for 30 multiple comparisons and maintain an experiment-wise α = 0.05, a Šidák corrected α = 0.0017 was used to assign statistical significance. In some cases, parameter pairs with moderate values of *r*, (|r|>0.4) failed to reach significance as a result of intrinsic variability inherent within this data. Such correlations are reported as moderate, and should be interpreted cautiously. Exact *r*, *R* ^2^, and *p* values are presented in Table 3.

**Table 2.** Basic properties of tSPNs

Property	Mean	SD	*n*	Min	Max
Membrane properties					
Resting membrane potential, mV	−59.8	6.8	35	−50	−80
Input resistance, MΩ	1044	576	34	246	2297
Input conductance, nS	1.31	0.84	34	0.44	4.1
Membrane time constant, ms	91.5	55.5	34	19	234
Capacitance, pF	89.1	26.6	34	51	157
Threshold					
Absolute voltage, mV	−42.4	6.2	35	−29.2	−58.8
Relative to V_hold_, mV	24.5	6.2	35	11.8	38.9
Measured rheobase, pA	27.5	16.5	35	5	70
Calculated rheobase, pA	30.7	18.2	34	10.1	95.9
AP					
Amplitude, mV	53.6	15.7	35	23.4	92.1
Peak, mV	11.2	16.9	35	−30.8	47.0
Half-width, ms	4.6	1.0	35	2.9	7.2
Rise slope, mV/ms	46.6	24.1	35	16.3	118
fAHP					
Amplitude, mV	15.0	3.7	24	6.7	21.1
Half-decay, ms	80.4	34.5	24	28.6	152
Duration, ms	229	68	24	109	363
sAHP					
Amplitude, mV	8.5	4.5	28	2.8	18.4
Half-decay, ms	342	211	27	101	1097
ƒ-I slope					
Maximal, Hz/pA	0.13	0.04	35	0.06	0.22
Sustained, Hz/pA	0.06	0.04	33	−0.16	0.11

Values of basic properties of tSPNs. SD, standard deviation; *n*, number of observations; Min, minimum value; Max, maximum value.

**Table 3.** Selected correlations between tSPN parameters

		*r*	*R* ^2^	*n*	*p*
Membrane properties					
R_in_	τ_m_	0.84	0.70	34	6.2 × 10^−10*^
R_in_	C_m_	−0.11	0.01	34	0.55
τ_m_	C_m_	0.40	0.16	34	0.020
Rheobase					
Calculated I_rheo_	Measured I_rheo_	0.82	0.68	34	2.0 × 10^−9*^
Calculated I_rheo_	V_hold_	−0.18	0.03	34	0.30
Calculated I_rheo_	g_in_ (R_in_^–1^)	0.85	0.72	34	3.0 × 10^−10*^
Calculated I_rheo_	τ_m_^–1^	0.72	0.52	34	1.5 × 10^−6*^
Calculated I_rheo_	C_m_	0.06	0.00	34	0.73
Firing frequency					
ƒ_max_ at 100 pA	R_in_	0.58	0.33	30	0.00086^*^
ƒ_sus_ at 100 pA	R_in_	0.21	0.05	26	0.29
ƒ_max_-I slope	R_in_	0.36	0.13	34	0.039
ƒ_max_-I slope	Calculated I_rheo_	−0.51	0.26	34	0.0023
ƒ_max_-I slope	τ_m_	0.14	0.02	34	0.43
ƒ_max_-I slope	C_m_	−0.31	0.10	34	0.073
ƒ_sus_-I slope	R_in_	0.29	0.09	32	0.10
ƒ_sus_-I slope	Calculated I_rheo_	−0.24	0.06	32	0.19
ƒ_sus_-I slope	τ_m_	0.26	0.07	32	0.15
ƒ_sus_-I slope	C_m_	−0.07	0.00	32	0.70
AHP					
fAHP half-decay	fAHP duration	0.84	0.70	24	3.0 × 10^−7*^
fAHP half-decay	R_in_	0.34	0.12	24	0.10
fAHP half-decay	C_m_	0.15	0.02	24	0.47
fAHP half-decay	τ_m_	0.43	0.18	24	0.036
fAHP half-decay	Calculated I_rheo_	−0.36	0.13	24	0.083
fAHP half-decay	ƒ_max_ at 2·I_min_	−0.66	0.43	19	0.0022
fAHP half-decay	ƒ_sus_ at 2·I_min_	−0.42	0.18	12	0.17
fAHP half-decay	ƒ_max_-I slope	−0.02	0.00	24	0.92
fAHP half-decay	ƒ_sus_-I slope	0.45	0.20	22	0.037
fAHP half-decay	sAHP half-decay	−0.38	0.14	19	0.11
sAHP half-decay	SRA ratio	0.65	0.42	27	0.00027^*^

Selected correlations reported in results. *r*, Pearson’s correlation coefficient; *R* ^2^, coefficient of determination; *n*, number of observations; *p* values calculated from two-tailed *t* test. Asterisk indicates statistically significant correlation at Šidák corrected α < 0.0017. R_in_, input resistance; τ_m_, membrane time constant; C_m_, membrane capacitance; I_rheo_, rheobase current; V_hold_, holding voltage; g_in_, input conductance; V_th_, threshold voltage; ƒ_max_, maximal IFR; ƒ_sus_, sustained firing rate; I_min_, minimal suprathreshold current.

## Results

### Thoracic ganglia composition

Postganglionic neurons have been shown to be either adrenergic or cholinergic (Jobling and Gibbins, 1999; Jänig, 2006). to assess neurotransmitter identity throughout the sympathetic chain, we used a choline-acetyltransferase (ChAT) transgenic mouse which fluorescently labels putative cholinergic postganglionic neurons (ChAT::eGFP), and co-immunolabelled tissue with an antibody to TH to label putative adrenergic neurons. Neurons were counted from stellate (T1 and T2) to T13 ganglia. We found that TH^+^ neurons comprised >97% of the population and no ganglion contained >6% presumptive cholinergic neurons. This agrees with prior findings in rodent thoracic ganglia which found few cholinergic neurons (Schäfer et al., 1998; Jobling and Gibbins, 1999; Masliukov and Timmermans, 2004; Schütz et al., 2015; see also Furlan et al., 2016). This indicates that cholinergic neurons in thoracic ganglia are rare. We therefore assume that a large majority of recorded cells were adrenergic.

As the majority of electrophysiological recordings focused on T5, diameter and number of TH-IR cells were examined in T5 ganglia in a separate sample from six adult mice (Fig. 1*C*). The mean number of TH-IR neurons counted in T5 ganglia was 247 ± 127 (ranging from 106 to 418). tSPNs had a mean cell diameter of 23.8 ± 5.4 μm with cell size distribution shown in Figure 1*D*. These values are smaller than those reported previously (31.0 ± 1.2 μm; cf. Jobling and Gibbins, 1999). Differing methodology may explain this discrepancy. The aforementioned study measured the diameter of dye-filled cells after microelectrode impalement, which would preferentially target larger neurons (Brown, 1981).

### Passive membrane properties

**Figure 2.**
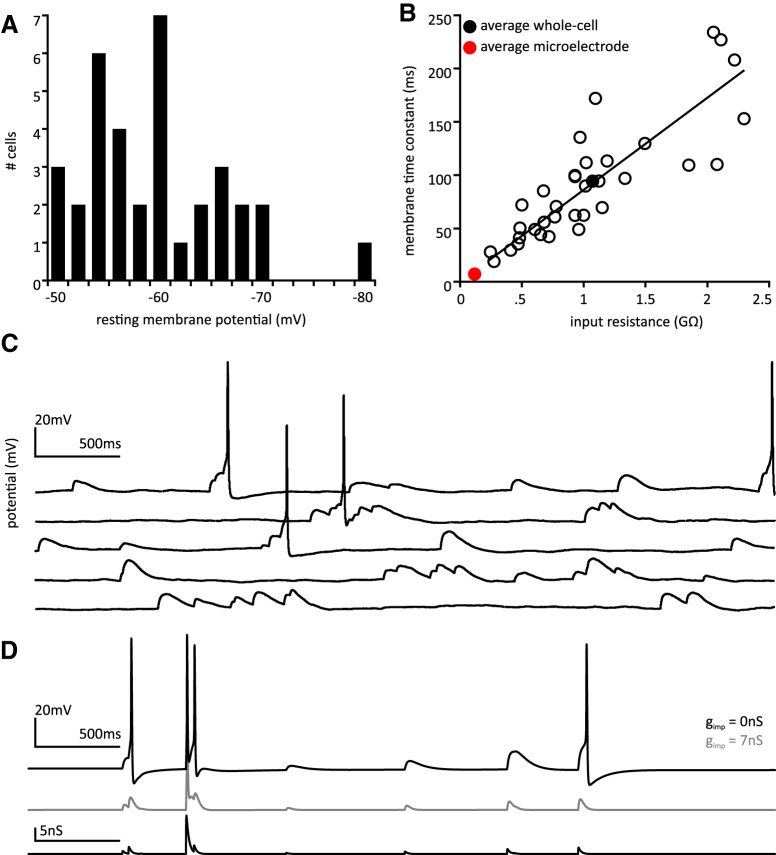
Passive membrane properties. ***A***, Histogram showing distribution of resting membrane potential values. ***B***, Input resistance is highly correlated with membrane time constant. Solid line indicates linear least-squares fit. Filled black circle represents population mean. Red filled circle represents population mean from (Jobling and Gibbins, 1999). ***C***, Example of synaptic summation leading to AP recruitment in a particularly active recording. Shown is a raster of epochs of spontaneous synaptic activity. Cell resting membrane potential was −60 mV. In this neuron, a τ_m_ of 109 ms led to comparably long EPSP membrane voltage decay τs. Vertical scale bar is 20 mV; horizontal scale bar is 500 ms. ***D***, top, Model neuron subjected to simulated synaptic input fires in response to synaptic summation. Middle, If an impalement conductance is added, synaptic summation is no longer effective. Bottom, Simulated g_syn_ used to generate voltage traces. Horizontal scale bar is 500 ms; vertical scale bars are 20 mV and 5 nS, respectively.

Whole-cell patch-clamp recordings were acquired from 35 tSPNs obtained from 30 adult mice. Basic cellular properties are summarized in Table 2. The distribution of resting membrane potential is shown in Figure 2*A*. Input resistance (R_in_) and membrane time constant (τ_m_) were, on average, an order of magnitude higher than values recorded using microelectrode recordings in mouse (Jobling and Gibbins, 1999; Fig. 2*B*) and guinea pig (Blackman and Purves, 1969) thoracic ganglia. R_in_ was strongly correlated with τ_m_ (Fig. 2*B*), but not cell capacitance (C_m_), an estimate of cell size. This indicates that membrane resistivity, but not cell size, is primarily responsible for the variability seen in resistance measures (Gustafsson and Pinter, 1984). C_m_ was also moderately correlated with τ_m_. A summary of correlation parameters is provided in Table 3.

One impact of larger τ_m_ is longer duration spontaneous EPSPs (sEPSPs) and consequently greater capacity for temporal summation. Spontaneous synaptic activity is often observed in whole-cell recordings, including instances of sEPSP summation that lead to recruitment of APs (Fig. 2*C*). In this neuron, a τ_m_ of 109 ms led to comparably long sEPSP membrane voltage decay τs.

To explore the impact of preserved passive membrane properties on synaptic summation, we implemented a synaptic conductance in the computational model. A template conductance was constructed with Poisson-distributed events whose amplitudes and mean frequency match values from whole-cell voltage clamp recordings. This template conductance was used to stimulate a standard model neuron (Fig. 2*D*, top trace) and a model neuron with simulated microelectrode impalement injury (middle trace). In the intact cell, synaptic events are larger in amplitude and synaptic summation can lead to cell recruitment. In the model neuron with simulated impalement, AP recruitment was observed only in response to the largest single synaptic event.

### Rheobase

The current required to depolarize a cell from its holding potential to firing threshold (rheobase) was examined in 35 cells by injecting long duration (1.5–3 s) pulses through patch electrodes. to control for the possible influence of a variable resting membrane potential on rheobase, tonic bias current was injected to hold cells at approximately −70 mV before rheobase estimation. Fluctuations in membrane voltage made it difficult to precisely set holding potential before injected current steps, and values ranged from −56 to −83 mV. We compared actual holding voltage against rheobase to determine whether this variability altered rheobase estimation. Rheobase was not correlated with holding potential.

**Figure 3.**
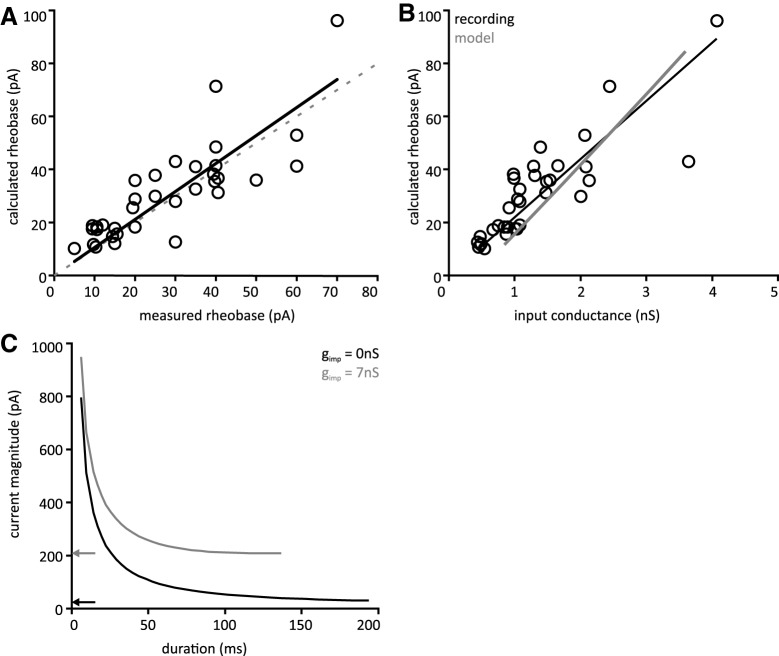
Factors affecting rheobase. ***A***, There is a strong correlation between measured rheobase and calculated rheobase and the two values are approximately equal. This suggests that rheobase is predominantly governed by ohmic phenomena. Dashed line is line of identity. ***B***, Calculated rheobase was well correlated with input conductance in recorded neurons, open circles. Gray line represents the rheobase versus input conductance relationship for a single model neuron chosen to fit experimental data. Standard model cell with G_M_ = 20 nS and G_A_ = 15 nS. ***A***, ***B***, Solid line represents least squares regression. ***C***, Strength-duration curves for model neurons. Black, standard model cell with no impalement conductance. Black arrow indicates rheobase. Gray, standard cell with g_imp_ = 7 nS, comparable to a microelectrode recording. Gray arrow indicates much higher rheobase for impaled cell.

Voltage threshold was assessed at minimal suprathreshold current intensity. Assuming cell depolarization is governed by Ohmic or non-rectifying processes, the ratio of relative voltage threshold to input resistance would predict rheobase (Gustafsson and Pinter, 1984). Indeed, measured and calculated rheobase are well correlated (Fig. 3*A*; Table 3) and approximately equal, indicating that rectifying currents do not play a major role in determining rheobase for the population. However, deviation of calculated rheobase values above and below those predicted by ohmic processes support a role for voltage-dependent conductances (Gustafsson and Pinter 1984). Values of measured and calculated rheobase are presented in Table 2. As calculated rheobase provides a more precise index of excitability, further analysis focuses on this parameter.

Calculated rheobase current was strongly correlated with input conductance, g_in_ = R_in_^−1^ (Fig. 3*B*), and moderately correlated with the inverse of time constant, τ_m_^−1^, but was uncorrelated with capacitance. A summary of correlation parameters is provided in Table 3.

We further investigated the relationship between input conductance and rheobase in a model cell. We adjusted g_leak_ to vary input conductance of a model neuron over most of the range observed in recorded neurons (0.5–3 nS). Bias current was adjusted to hold the model cell at −70 mV. The rheobase was then calculated for each value of input conductance by using a binary search algorithm to find the minimal injected current which produces a spike (Fig. 3*B*, gray line). The results show that there is a deterministic relationship between rheobase and input conductance that can help to explain some of the correlation observed in recorded neurons. However, given the variability of rheobase measures in recorded cells with comparable values for input conductance, it is clear that input conductance alone does not fully explain the range of rheobase values observed in recorded neurons.

Rheobase values were 80–90% lower than values estimated in tSPNs previously with microelectrode recordings in both mouse and guinea pig (Blackman and Purves, 1969; Jobling and Gibbins, 1999). Reduced rheobase values indicate that tSPNs are much more excitable than previously considered. To more fully explore the influence of microelectrode impalement on cell excitability, we constructed strength-duration curves for model cells. In a standard model cell, the strength-duration curve follows a characteristic inverse curve. After implementation of an impalement conductance consistent with a microelectrode recording, rheobase is increased ∼8-fold (Fig. 3*C*). This is consistent with the discrepancy between our experimental findings and the aforementioned studies using microelectrodes.

### Repetitive firing

**Figure 4.**
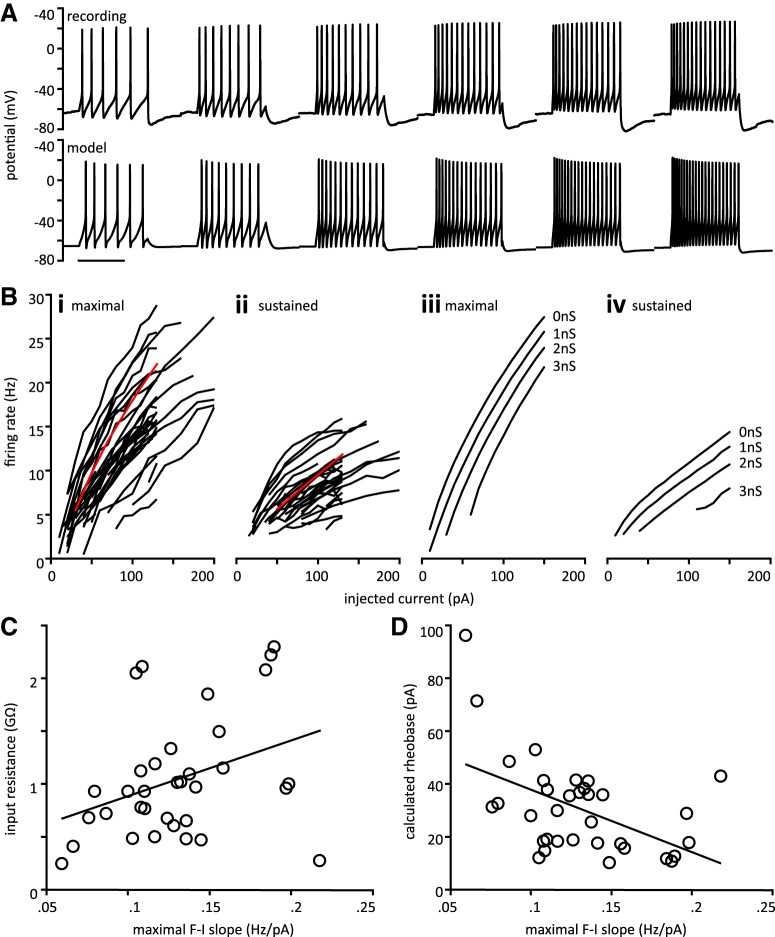
tSPNs exhibit repetitive firing. ***A***, top, Representative trace from a tSPN showing increases in repetitive firing frequency in response to increasing current steps. Bottom, Model neuron also showing repetitive firing. Standard model with G_M_ = 30, G_KCa_ = 70, G_A_ = 80, G_leak_ = 2 nS. Injected current from left to right in both recorded neuron and model is 30, 50, 70, 90, 110, 130 pA. Scale bar is 1 s. ***B***, ƒ-I relations for recorded and model neurons. ***i***, Maximal IFR is plotted versus injected current for all cells. ***ii***, Same as ***Ci*** with sustained firing rate. Red line in i and ii is maximal and sustained ƒ-I curve from model neuron in ***A***. ***iii***, Maximal ƒ-I curve from a model neuron in which g_leak_ was adjusted from 0 to 3 nS. Note: as varying input conductance also changes holding potential, each model neuron was subjected to a different holding current to hold the initial voltage at –70 mV. Also note that g_leak_ is distinct from g_imp_. ***iv***, Corresponding sustained ƒ-I curves. Model parameters other than g_leak_ are the same as in ***A***. ***C***, Maximal ƒ-I slope is positively correlated with input resistance. ***D***, Maximal ƒ-I slope is negatively correlated with calculated rheobase.

Increasing current steps were delivered to assess repetitive firing properties from a holding potential of approximately −70 mV. All cells (*n* = 35) were capable of repetitive firing in response to sustained current injection. This contradicts an earlier report that tSPNs fire phasically in response to depolarization (Jobling and Gibbins, 1999). Figure 4*A* shows an example of a recorded cell which fires repetitively at progressively higher frequency in response to increasing depolarizing current steps (top). A model neuron that used known voltage-dependent conductances for paravertebral sympathetic neurons and incorporated values for input conductance obtained from our whole-cell recordings was able to replicate repetitive firing (Fig. 4*A*, bottom).

ƒ-I relations were obtained by plotting the maximal (initial) and sustained firing rate versus injected current magnitude. Figure 4*Bi*,*ii* shows the maximal and sustained, respectively, ƒ-I curves for all cells. Maximal IFR did not exceed 28 Hz, while sustained firing rate did not exceed 17 Hz for the highest steps given. ƒ-I curves were approximately linear. to determine the role that input conductance plays in determining ƒ-I relations in tSPNs, we selected a model neuron that matches the experimental ƒ-I curves and then systematically changed input conductance by varying g_leak_ from 0 to 3 nS. Of note, varying input conductance also changes holding potential so to remain consistent with experimental protocol, each model neuron was subjected to a different holding current to hold the initial voltage at −70 mV. Figure 4*Biii*,*iv* demonstrates that altering g_leak_ can shift the ƒ-I curve, but it does not appear to change the ƒ-I slope. Thus, input conductance cannot fully account for the range of ƒ-I curves observed. To determine whether other model parameters are capable of changing ƒ-I slope, we systematically varied each parameter and observed its influence on maximal and sustained ƒ-I curves. Most notably, C_m_ appears to influence the slope of the maximal ƒ-I curve while G_CaL_ and G_KCa_ impact the slope of the sustained ƒ-I curve. Other model parameters (G_Na_, G_K_, G_M_, G_A_, G_leak_) are able to shift ƒ-I curves without significantly altering slope. Thus, we are able to match any realistic ƒ-I curve by adjusting model parameters, which implies that a host of intrinsic cellular properties are responsible for the range of ƒ-I curves we observed.

Slope for both maximal and sustained ƒ-I curves was calculated as a measure of excitability (Zimmerman and Hochman, 2010). In short, a cell with a higher ƒ-I slope would respond to an incremental change in current with a higher change in firing frequency. In this way, ƒ-I slope can be thought of as the gain between input and output of a neuron. Values for maximal and sustained ƒ-I slope are given in Table 2.

We assessed the role of variations in R_in_ and rheobase in cell excitability based on ƒ-I slope measures. Maximal firing rate at 100-pA current injection was significantly correlated with R_in_. Maximal ƒ-I slope was moderately correlated with R_in_ (Fig. 4*C*), and moderately and negatively correlated with calculated rheobase (Fig. 4*D*). No such relationship was found for τ_m_ or C_m_. A summary of correlation parameters is provided in Table 3. Cells with lower rheobase and higher R_in_ had higher ƒ-I slopes, suggesting that Ohmic properties contribute to the ƒ-I response.

### Impalement simulation

**Figure 5.**
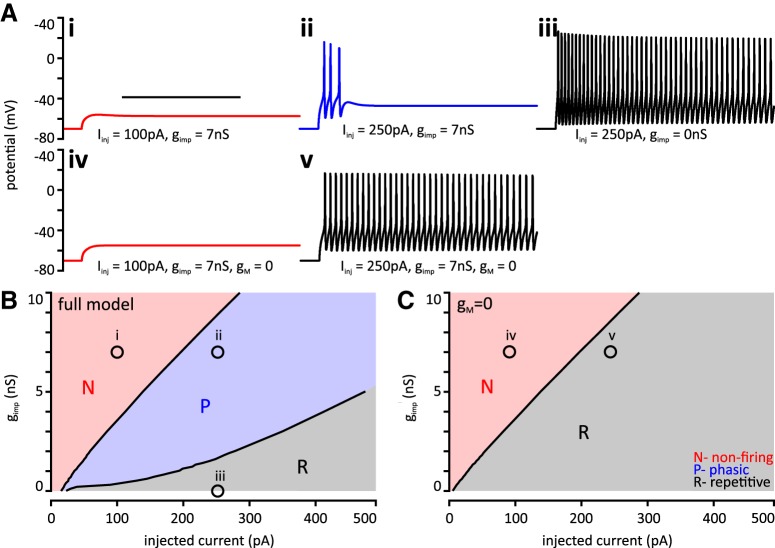
Simulated impalement can alter firing properties. ***A***, Impact of injected current and impalement conductance, g_imp_, on firing properties. Voltage response of a model cell to subthreshold (***i***) and suprathreshold (***ii***) current injection after g_imp_ is set to 7 nS, analogous to a microelectrode recording. Only phasic firing is observed. ***iii***, Repetitive firing is observed when g_imp_ is set to 0 nS, analogous to a whole-cell recording. When g_M_ is removed from the model, the same parameters used in ***i***, ***ii*** lead to non-firing (***iv***) and repetitive firing (***v***). ***B***, Shaded regions indicate the set of all parameters which lead to non-firing (N, red), phasic firing (P, blue), and repetitive firing (R, black/gray). At g_imp_ = 0 nS, the model neuron transitions rapidly from N to R, and repetitive firing results from any current injection above ∼20 pA. At g_imp_ = 7 nS, the model neuron transitions from N to P at around 200-pA current injection, and repetitive firing is not observed for injected current less than 500 pA. ***C***, same as ***B*** with g_M_ set to 0 nS. Removing I_M_ from the model by setting g_M_ = 0 nS eliminates phasic firing altogether, i.e., cells transition directly from N to R regardless of impalement conductance. Open circles in ***B***, ***C*** indicate the g_imp_ and injected current values used to generate traces in ***A***. Standard model with g_leak_ = 0.5 nS.

The discrepancy between observations of phasic and repetitive firing likely arises as a result of leak introduced by microelectrode impalement (Springer et al., 2015). We undertook additional modeling to test whether an impalement injury can convert repetitive to phasic firing. An additional impalement conductance, g_imp_, was added to a standard model cell. Reversal potential of g_imp_ was set at −15 mV. We explored the relationship between g_imp_ and firing type over a range of conductance and current injection combinations (Fig. 5). For a given set of g_imp_ and injected current, a model cell was characterized as non-firing (N), phasic firing (P), or repetitively firing (R). Setting g_imp_ to 7 nS results in an input resistance of ∼100 MΩ, the mean value of input resistance reported by Jobling and Gibbins (1999). With this level of microelectrode leak, non-firing was observed in response to subthreshold current injection (Fig. 5*Ai*), and phasic firing was observed in response to suprathreshold current injection over the range of values tested by Jobling and Gibbins (Fig. 5*Aii*). However, when g_imp_ was set at 0 nS, analogous to a whole-cell recording, repetitive firing was observed instead (Fig. 5*Aiii*).

Prior studies have reported that phasic firing sympathetic neurons could instead fire repetitively if I_M_ was blocked (Brown and Adams, 1980; Cassell et al., 1986). To test this, we blocked I_M_ in our model cell by setting g_M_ to 0 nS. This change completely eliminated phasic firing in the model, and only non-firing (Fig. 5*Aiv*) or repetitive firing was observed (Fig. 5*Av*).

To more extensively characterize this phenomenon, the boundaries between each of the three firing types were identified using a binary search algorithm. In the case where I_M_ is included in the model (Fig. 5*B*), there was a rapid transition from repetitive to phasic firing as g_imp_ is increased. When I_M_ is removed (Fig. 5*C*), the phasic firing region (P) disappears.

### SRA

**Figure 6.**
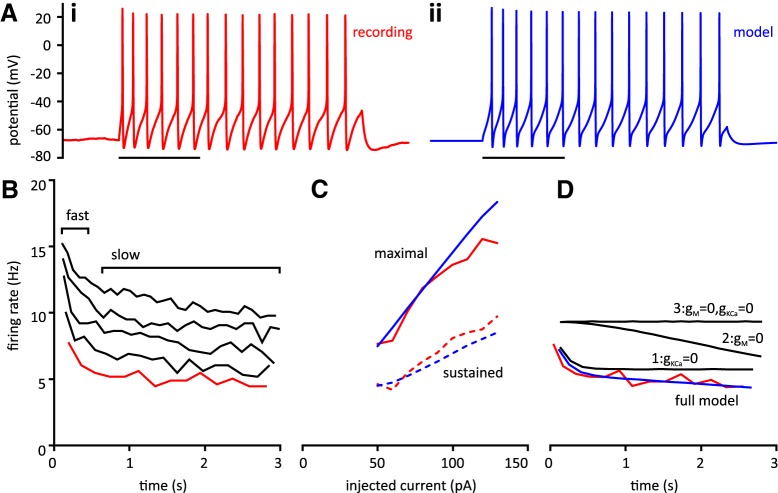
Modeling suggests that SRA in tSPNs depends on both I_M_ and I_KCa_. ***A*i**, Representative trace showing tSPN response to 50-pA current injection. Note that the interspike interval increases over time, corresponding to a decrease in instantaneous frequency. ii, Trace from a model cell chosen to fit the recording shows similar SRA for 50-pA current injection. Maximal conductances are: G_Na_ = 400 nS, G_K_ = 3000 nS, G_CaL_ = 1.2 nS, G_M_ = 40 nS, G_KCa_ = 60 nS, G_A_ = 80 nS, G_H_ = 1 nS, G_leak_ = 2 nS. Scale bar in both panels is 1 s. ***B***, Instantaneous frequency versus time for the same recorded cell at 50-, 70-, 90-, 110-, and 130-pA current injection (from bottom to top). The 50-pA curve (red) corresponds to the trace in ***Ai***. Fast and slow components of adaptation are indicated. ***C***, Maximal and sustained ƒ-I curves match well between recorded and modeled cell over a range of injected currents. Red, maximal (top, solid) and sustained (bottom, dashed) ƒ-I curves for the cell in ***Ai***, ***B***. Blue lines are the corresponding ƒ-I curves from the model cell in ***Aii***. ***D***, Instantaneous frequency versus time curves for the model cell in ***Aii***. The recorded 50-pA curve from ***B*** is reproduced for comparison to the analogous curve generated in the model cell in ***Aii*** (blue). Black curves numbered 1–3 represent effect of removal of two conductances from the model. Removal of g_KCa_ (curve 1) predominantly influences the slow SRA. Removal of g_M_ (curve 2) predominantly influences the fast SRA. Removal of both (curve 3) eliminates SRA. The ordinate axis is shared among ***B–D***.

Implicit in the observation that sustained firing rates were lower than initial observed frequencies is that all cells displayed SRA, or a decrease in firing rate over time. We were able to replicate SRA in our model (Fig. 6*A*). The time course of adaptation consists of a fast and a slow phase (Fig. 6*B*).

The difference between the initial firing rate and the sustained firing rate becomes more pronounced as injected current is increased in all cells. This can be illustrated by comparing the maximal ƒ-I curve to the sustained ƒ-I curve in both recorded and model neuron over a range of current injection (Fig. 6*C*). This relationship between maximal and sustained firing rate is a common feature of adapting neurons (Benda and Herz, 2003).

Several mechanisms have been proposed to underlie SRA in different neuronal populations including Na^+^ channel inactivation (Miles et al., 2005), fAHP summation (Powers et al., 1999), activation of I_KCa_ (Miles et al., 2005) and activation of I_M_ (Yi et al., 2015). We selectively removed conductances from the model and determined which were primarily responsible for SRA (Fig. 6*D*). Removal of I_KCa_ preferentially impaired the later phase of adaptation (curve 1) while removal of I_M_ preferentially impaired the early phase of adaptation (curve 2). Removal of both I_KCa_ and I_M_ completely eliminated SRA (curve 3). Our model supports the conclusion that the combination of these conductances is necessary to replicate SRA.

### AHP

**Figure 7.**
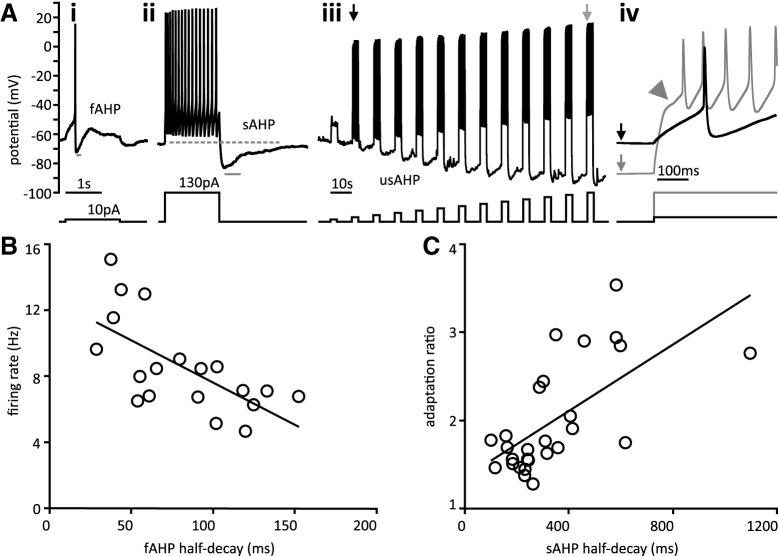
AHP. ***A***, Side by side comparison of three types of AHPs. ***i***, fAHP present after single spike. ***ii***, sAHP is present in the same cell only after repetitive firing. The half-decay time of fAHPs and sAHPs are indicated by the gray bar beneath each trace. Scale bar 1 s. ***iii***, Depolarizing current steps (10–130 pA in 10-pA increments) in a different cell showing the progressive hyperpolarization characteristic of the usAHP. Scale bar 10 s. ***iv***, Expanded view of voltage traces in ***Aiii*** indicated by vertical arrows. Note that the gray trace is hyperpolarized by 20 mV compared to the black trace and has a characteristic notch (arrowhead) on depolarization. Current injection profile is shown below each trace. ***B***, fAHP half-decay was negatively correlated with maximal firing rate at twice the minimal suprathreshold current. ***C***, SRA ratio is positively correlated with sAHP half-decay. Black line is the linear regression.

AHP dynamics play an important role in regulating neuronal firing. Based on decay time, we identified three types of AHP within the thoracic ganglia. These include the fAHP after a single AP, and the sAHP and ultra-slow AHP (usAHP) after multiple APs (Fig. 7*A*).

Fast post-spike after-hyperpolarization (fAHP) amplitude, half-decay time, and duration were measured at rheobase current injection (Fig. 7*Ai*). Parameters related to fAHP are summarized in Table 2. Half-decay time was very well correlated with duration and more reliably obtained, so further analysis focused on fAHP half-decay time. fAHP half-decay time was compared to passive membrane properties and rheobase. fAHP half-decay time was not correlated with R_in_, C_m_, or rheobase, but was moderately correlated with τ_m_. Previous studies have reported an inverse relationship between fAHP duration and firing rate in motoneurons (Brownstone et al., 1992; Stauffer et al., 2007). To determine whether this relationship exists in postganglionic neurons, we plotted fAHP half-decay time versus sustained firing rate at two times the minimal suprathreshold current injection. Note that this corresponds to twice the current magnitude used to estimate fAHP half-decay time. We found that there is indeed a moderate negative correlation between fAHP half-decay time and maximal firing rate (Fig. 7*B*). fAHP half-decay time was also moderately correlated with sustained ƒ-I slope but not ƒ-I slope.

sAHPs were also observed following larger depolarizing steps that elicited higher repetitive firing frequencies (Fig. 7*Aii*). Only cells displaying obvious sAHP were analyzed (*n* = 27 of 35). sAHPs were measured at maximal current injection. Parameters related to sAHP are summarized in Table 2. sAHP half-decay time was four-fold longer on average than fAHP half-decay, but the two were not correlated. To examine the relationship between sAHP and SRA, we plotted the sAHP half-decay versus the SRA ratio for 27 cells (Fig. 7*C*). We found the two parameters were significantly correlated. A summary of correlation parameters for both fAHP and sAHP is provided in Table 3. As with SRA, our computational model showed that I_M_ and I_KCa_ were capable of reproducing sAHP after repetitive firing (not shown).

Prior work in the rabbit SCG identified a long-lasting AHP following sustained depolarization that was due to the ouabain sensitive Na^+^/K^+^-ATPase (Lees and Wallis, 1974). In the neonatal mouse spinal cord, it has been shown to be due to activation of α3 Na^+^/K^+^-ATPase (Picton et al., 2017).This AHP is unique in its ability to hyperpolarize a cell membrane beyond the reversal potential of K^+^. We identified an AHP with a similar time course. We injected depolarizing current to cause the cell to fire repetitively. This repetitive firing led to a steadily increasing hyperpolarization (Fig. 7*Aiii*). In the example shown, induced epochs of repetitive firing led to a 20-mV membrane hyperpolarization (Fig. 7*Aiv*). This feature was present in two of 14 cells tested with a current step protocol that would allow for its observation. Of note, the usAHP was observed only in relatively high resistance cells when ATP and GTP were included in the electrode solution. This AHP was also able to achieve a membrane potential of −101.7 ± 11.5 mV, which is more negative than the calculated −98 mV K^+^ reversal potential. The time course of this hyperpolarization is too long to be due to I_M_ or I_KCa_.

### Subthreshold conductances

Subthreshold conductances can play an important role in determining cell excitability and firing properties. We evaluated activation of these conductances with current steps that included assessment at hyperpolarized membrane potentials seen during the usAHP.

**Figure 8.**
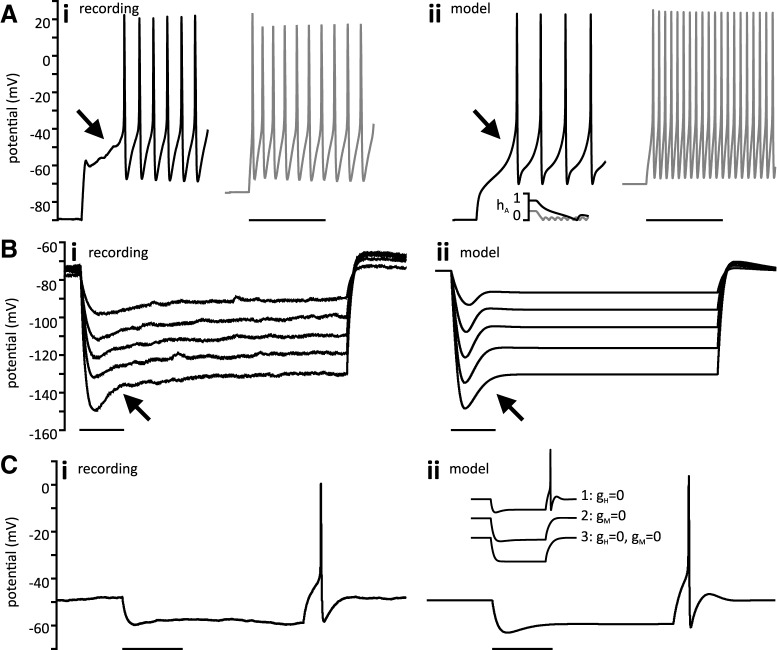
Subthreshold conductances. ***Ai***, A cell depolarized from −90 mV exhibits a characteristic notch (arrow) accompanied by a delay in spiking (black trace). The same cell depolarized from −70 mV does not have a notch (gray trace). ***ii***, Model neuron showing comparable results with pre-spike inflection seen only for hyperpolarized trace. Standard model with G_M_ = 10 nS, G_KCa_ = 10 nS, G_A_ = 90 nS, G_leak_ = 0 nS. Inset, Magnitude of h_A_ at onset of current injection shows that I_A_ is less inactivated (h_A_ is higher) at hyperpolarized voltage, and I_A_ takes longer to fully inactivate. Scale bars represent 500 ms for all panels. ***Bi***, Voltage sag, indicated by arrow, on hyperpolarization beyond −90 mV in a cell held at −70 mV. Note that the effect becomes more pronounced with greater hyperpolarization. ***ii***, Model neuron showing similar sag. Standard model with G_A_ = 5 nS and G_leak_ = 0.1 nS. ***Ci***, Hyperpolarizing trace from a different cell held at −50 mV showing rebound spiking associated with voltage sag. ***ii***, Model neuron showing rebound spiking at the same holding voltage and current injection. Maximal conductances are: G_Na_ = 200 nS, G_K_ = 2000 nS, G_CaL_ = 1.2 nS, G_M_ = 20 nS, G_KCa_ = 20 nS, G_A_ = 20 nS, G_H_ = 1 nS, G_leak_ = 2 nS. Removal of g_H_ (curve 1) does not inhibit rebound firing. Removal of g_M_ eliminates firing (curve 2) as does removal of both currents (curve 3).

In response to depolarizing current steps, membrane voltage first followed an exponential time course with subsequent recruitment of voltage-gated conductances that altered the trajectory. In 24 of 35 cells, membrane trajectory exhibited a negative deflection from the exponential trajectory which preceded activation of voltage gated Na^+^ conductance. The observed deflection, or “notch,” in membrane led to a delay in the first AP in a train (Fig. 8*Ai*) and has been described previously in tSPNs (Jobling and Gibbins, 1999). This phenomenon was often observed at a holding potential of −70 mV, and became more pronounced with greater hyperpolarization (−90 mV). This is consistent with activation of the transient, voltage-gated A-type K^+^ current (I_A_). To test the contribution of I_A_ to the notch and delayed firing, we held a model neuron at two different holding potentials and found that the change in trajectory was indeed attributable to de-inactivation of I_A_ (Fig. 8*Aii*; Rush and Rinzel, 1995). Notably, a similar notch was observed in cells displaying usAHP (Fig. 7*Aiv*), demonstrating that the usAHP leads to a state of membrane hyperpolarization where I_A_ would delay onset of firing.

During hyperpolarizing current injection, a depolarizing voltage “sag” was often observed. When present, a voltage sag was easily detected with membrane hyperpolarization beyond −100 mV (Fig. 8*Bi*) but was also observed at less negative hyperpolarization (Fig. 8*Ci*). We found a voltage sag in 17 of 28 cells hyperpolarized to at least −100 mV from a holding potential of −70 mV. This phenomenon has been previously reported in mouse tSPNs (Jobling and Gibbins, 1999) and other mammalian sympathetic neurons (Cassell et al., 1986) where it has been attributed to the anomalous rectifier, or H-current (I_H_). To support a role for I_H_, this conductance was implemented in the computational model and was found to reproduce the observed voltage sag (Fig. 8*Bii*). I_H_ has also been shown to contribute to a more depolarized membrane potential (Pape, 1996; Lamas, 1998), so we compared resting membrane potential in cells with (*n* = 17) and without (*n* = 11) evidence of I_H_ but found no significant differences (Student’s *t* test, two-tailed, *p* = 0.17).

I_H_ has also been implicated in post-inhibitory rebound firing (Pape, 1996; Ascoli et al., 2010; Engbers et al., 2011; Ferrante et al., 2017). Sag was seen in 12 of 13 tSPNs exhibiting rebound firing, but rebound firing was only observed when cells were held closer to firing threshold (between −60 and −50 mV; Fig. 8*Ci*) where I_M_ has been shown to be responsible for inducing a voltage sag and rebound firing (Constanti and Galvan, 1983). We used a computational model to understand the relative contributions of I_H_ and I_M_ and determined that sag is due to I_H_ for significant hyperpolarizations, and I_M_ for more moderate hyperpolarization. Rebound firing can occur in the absence of I_H_ but does not occur in the absence of I_M_ following release from moderate (∼10 mV) hyperpolarization, indicating that I_H_ is neither necessary nor sufficient to induce rebound firing in tSPNs (Fig. 8*Cii*).

## Discussion

### Reappraisal of physiologic consequence of passive membrane properties

We obtained high-quality recordings of mouse tSPNs and built a computational model to provide mechanistic insight into their function. Whole-cell recordings preserve membrane properties and provide an accurate representation of tSPN function. This is critically important, as the impalement conductance introduced by microelectrode recordings can change passive membrane properties (Staley et al., 1992; Cymbalyuk et al., 2002; Springer et al., 2015), reduce apparent excitability, underestimate the importance of synaptic convergence (Karila and Horn, 2000; Horn and Kullmann, 2007), and prevent repetitive firing (Springer et al., 2015).

Input resistance (R_in_) and membrane time constant (τ_m_) were highly correlated and their values, as well as rheobase, occupy an approximately 10-fold range. Values of R_in_ and τ_m_ are an order of magnitude larger than values previously obtained from the same population using traditional microelectrode recordings (Blackman and Purves, 1969; Jobling and Gibbins, 1999), which indicates that the excitability of tSPNs has been substantially underestimated. Measured cell diameters in the T5 ganglion occupied a five-fold range (cf. Jobling and Gibbins, 1999). Capacitance (C_m_) values occupied a three-fold range, and were unrelated to cell recruitment. The strong observed relationship between R_in_ and measures of firing threshold (i.e., rheobase) demonstrate that ohmic processes dominate tSPN recruitment. These observations suggest that membrane resistivity rather than cell size is the primary determinant of recruitment threshold across the population (Gustafsson and Pinter, 1984), although it is unclear whether the observed variability in excitability represents a population recruitment principle.

The preservation of the passive membrane electrical properties R_in_ and τ_m_ leads to synaptic events of greater amplitude and longer duration, which has important consequences for synaptic recruitment. Paravertebral neurons receive nicotinic EPSPs comprising both sub- and suprathreshold events of variable amplitude (Nishi and Koketsu, 1960; Blackman and Purves, 1969; Karila and Horn, 2000; Bratton et al., 2010). An overall increase in EPSP amplitude would convert many subthreshold events into suprathreshold events, thereby increasing tSPN firing rate (Bratton et al., 2010). Traditionally, summation of EPSPs was not thought to contribute to cell recruitment in paravertebral ganglia (North, 1986; McLachlan et al., 1997; Jänig, 2006). However, recent whole-cell recordings from rat SCG demonstrate long-duration sEPSPs with much greater capacity for summation (Springer et al., 2015). We also observed long-duration sEPSPs with decay time constant comparable to τ_m_ and examples of sEPSP summation leading to cell recruitment. This provides direct support for the gain hypothesis for amplification of preganglionic activity (Karila and Horn, 2000; Horn and Kullmann, 2007). The observed τ_m_ values indicate that tSPNs could act as integrators during states of strong preganglionic sympathetic drive from individual neurons (Jänig, 1985; Ivanov and Purves, 1989) and could widen the temporal window for coincidence detection and summation of convergent synchronous preganglionic inputs (Skok, 1973; König et al., 1996; Ratté et al., 2013). These observations support the concept that tSPNs do not merely relay preganglionic activity, but rather actively integrate and amplify sympathetic output. Metabotropic receptor-mediated changes in intrinsic membrane conductances may further amplify this process (North, 1986; Karila and Horn, 2000).

Additionally, important was the observation that all tSPNs were capable of firing repetitively, which contrasts traditional observations in all paravertebral neurons, including tSPNs, of phasic firing in response to sustained current injection (Jobling and Gibbins, 1999; Jänig, 2006; Springer et al., 2015). Recent whole-cell recordings in rat SCG similarly found paravertebral neurons were capable of repetitive firing, and suggested the discrepancy was a result of impalement conductance (Springer et al., 2015). We were able to replicate these results using our model; by introducing an impalement conductance consistent with microelectrode impalement, we were able to convert repetitively firing model neurons to phasically firing model neurons. Phasic firing after impalement injury appears to be dependent on the presence of I_M_, as blocking I_M_ can convert sympathetic neurons from phasic to repetitively firing (Brown and Adams, 1980; Brown and Constanti, 1980; Cassell et al., 1986; Luther and Birren, 2009). This observation was reproduced by subtracting I_M_ in our model. I_KCa_ has also been shown to contribute to the interconversion of sympathetic neuron membrane firing properties (Sacchi et al., 1995; Luther and Birren, 2009). Thus, the firing properties of paravertebral sympathetic neurons that exhibit I_M_ and I_KCa_ are particularly sensitive to impalement leak, which underscores the importance of using whole-cell recordings. Blackman and colleagues were able to observe repetitive firing with microelectrodes, a finding that has been consistently overlooked (Blackman and Purves, 1969). A possible explanation could be differences in ion channel expression between the mouse and guinea pig.

### The physiologic relevance of repetitive firing in tSPNs

The physiologic relevance of repetitive firing in tSPNs in response to current stimulation might be dismissed if one assumes that postganglionic neurons are only driven by nicotinic preganglionic input. However, paravertebral neurons can exhibit long-lasting depolarization and sustained firing (Blackman and Purves, 1969; Jänig et al., 1982; Kawatani et al., 1987). Activation of metabotropic muscarinic and various other non-cholinergic receptors are implicated (Jänig et al., 1982; North, 1986; Kawatani et al., 1987; Elfvin et al., 1993). These studies support the idea that tSPNs can generate sustained sympathetic drive with limited influence from preganglionics.

Passive membrane properties and various conductances are responsible for sculpting the firing response of tSPNs. R_in_ is important in determining firing rate over a range of injected current values. R_in_ also impacts the slope of the ƒ-I curve. tSPNs with steeper slope may be more effective at amplifying postganglionic output gain (Salinas and Thier, 2000; Zimmerman and Hochman, 2010). Given the relatively low steady-state firing rates of preganglionic neurons observed *in vivo* (Jänig, 2006), the physiologic relevance of variability in response amplification is unclear (Springer et al., 2015).

However, synaptic drive may contribute to response amplification during bouts of metabotropic receptor-mediated sustained activity described above. We observe spontaneous EPSCs with amplitudes ranging from 10 pA to over 100 pA (data not shown). Comparing these amplitudes to values of rheobase (range, 5–70 pA) supports conditions where synaptic actions are capable of transient response amplification.

### Relating observed cellular properties to underlying conductances

While the firing rate of tSPNs is strongly determined by the temporal dynamics of the fAHP, a feature carried by I_A_ and I_K_ in rodent SCG (Belluzzi and Sacchi, 1988), the mechanisms underlying SRA have not been studied in paravertebral ganglia including tSPNs. SRA has been well characterized elsewhere (Benda and Herz, 2003; Benda and Tabak, 2013). Contributions from I_M_ and I_KCa_ are among the proposed mechanisms (Sawczuk et al., 1997; Powers et al., 1999; Miles et al., 2005; Yi et al., 2015), and these currents have been previously identified in rodent paravertebral ganglia (Sacchi et al., 1995; Davies et al., 1996; Haley et al., 2000; Locknar et al., 2004; Maingret et al., 2008). Our modeling found that I_M_ and I_KCa_ were required to replicate the fast and slow components of SRA, respectively. I_M_ and I_KCa_ are also known to contribute to the sAHP in rodent SCG and hippocampus (Storm, 1990; Sacchi et al., 1995), and inclusion of I_KCa_ or I_M_ in the model reproduced the sAHP after repetitive firing. That SRA ratio and sAHP half-decay were correlated further supports co-involvement of these conductances.

### Other factors contributing to modulation of tSPN excitability

tSPNs are known to express I_A_, I_H_ and I_M_ (Jobling and Gibbins, 1999). These currents have been shown to modulate EPSP amplitude, synaptic integration, membrane potential, and repetitive firing rate (Connor and Stevens, 1971; Storm, 1990; Rush and Rinzel, 1995; Hoffman et al., 1997; Lamas, 1998; Prescott et al., 2006; George et al., 2009; Kullmann et al., 2016). We found evidence of I_A_, I_H_ and I_M_ in our recordings by observing phenomena such as notch, sag, and rebound firing, and we replicated their effects using computational modeling. These phenomena typically require hyperpolarization to emerge. While there are no known inhibitory synapses in sympathetic ganglia (McLachlan, 2007), a slow IPSP due to metabotropic activation of K^+^ conductances has been observed in SCG (Libet and Kobayashi, 1974; North, 1986). Another method of hyperpolarization observed in a small group of tSPNs is the slowly developing usAHP that follows prolonged activity (Zhang and Sillar, 2012). The usAHP has been observed in rabbit SCG (Lees and Wallis, 1974) and reflects Na^+^-dependent activation of the ouabain-sensitive α3 Na^+^/K^+^-ATPase (Picton et al., 2017). These long-lasting hyperpolarizations may provide a physiologic mechanism by which the aforementioned phenomena may emerge.

10.1523/ENEURO.0433-18.2019.ed1 Extended Data 1: Computational model code Python and MATLAB code for the computational model of tSPN. Documentation is provided within the code. Download Extended Data 1, ZIP file.

## Synthesis

Reviewing Editor: Upinder S. Bhalla, National Centre for Biological Sciences, Tata Institute of Fundamental Research; Decisions are customarily a result of the Reviewing Editor and the peer reviewers coming together and discussing their recommendations until a consensus is reached. When revisions are invited, a fact-based synthesis statement explaining their decision and outlining what is needed to prepare a revision will be listed below. The following reviewer(s) agreed to reveal their identity: Rohit Manchanda. Note: If this manuscript was transferred from JNeurosci and a decision was made to accept the manuscript without peer review, a brief statement to this effect will instead be what is listed below.

The reviewers and I discussed the revised version of this MS and we agreed

that it was substantially improved, and the authors had addressed almost all

points raised by the reviewers. There is one minor but essential revision

that the authors should do:

The authors should drop the classification of cells into three types, as this

is neither convincing nor essential to the point of the paper.

For the author's benefit, here are the specific reviewer's comments:

“One point that is not addressed satisfactorily is their classification of these cells into three types based on the peak voltage of the initial spike compared to the peak voltage of subsequent spikes. They state that other AP features were different but none of the basic membrane properties correlated with these features. There is no indication of any functional significance of this classification. It seems to be just as good a way to quantitavely classify neurons as any number of other AP parameters. Moreover, in Figures 7 C and D the data points suggest a continuum rather than discrete groups. Doing a PCA and clustering showing clearly separate clusters would have been more convincing. It is not clear what value this section adds to the study.”
